# Clinical response to nonsurgical periodontal therapy is associated with decreased serum leukocyte count and uric acid levels in kidney transplant recipients*

**DOI:** 10.1590/1678-7757-2024-0206

**Published:** 2024-09-30

**Authors:** Samira Vasconcelos Gomes, Danila Lorena Nunes-Dos-Santos, Luciana Salles Branco-De-Almeida, Bruno Braga Benatti, Vandilson Rodrigues

**Affiliations:** 1 Universidade Federal do Maranhão Departamento de Odontologia São Luís Brasil Universidade Federal do Maranhão, Departamento de Odontologia, São Luís, Brasil.; 2 Universidade Federal do Maranhão Hospital Universitário São Luís Brasil Universidade Federal do Maranhão, Hospital Universitário, São Luís, Brasil.

**Keywords:** Periodontal disease, Nonsurgical periodontal therapy, Kidney transplantation, Biomarker

## Abstract

**Objective:**

This study sought to investigate the relationship between clinical response to nonsurgical periodontal therapy (NSPT) and serum changes in leukocyte count, fasting blood glucose, hemoglobin, hematocrit, creatinine, and uric acid in kidney transplant recipients (KTR).

**Methodology:**

A prospective study was performed on 20 KTRs. Periodontal and serum data were collected before and 90 days after NSPT, and delta values (Δ = after NSPT - before) were calculated. Periodontal assessment included periodontal probing depth (PPD), clinical attachment level (CAL), and bleeding on probing (BOP). Patients were classified based on the presence of periodontitis and then categorized into stages.

**Results:**

Patients showed a reduction in the percentage of sites with PPD≥3mm, PPD≥4 mm and BOP, after NSPT. There was a direct correlation between the deltas of leukocyte count and CAL ≥3 mm (r=0.645, P=0.002) and BOP (r=0.663, P=0.001), and the deltas of uric acid and CAL ≥3 mm (r=0.562, P=0.010).

**Conclusion:**

A good clinical response to NSPT may affect the reduction of serum levels of leukocyte count and uric acid, suggesting a beneficial effect on systemic health in KTR.

## Introduction

Chronic kidney disease (CKD) is one of the leading causes of death and increased morbidity in the 21^st^ century, affecting over 800 million people worldwide, due in part to the global increase in associated risk factors such as obesity, diabetes, and hypertension.^1^ Kidney transplantation (KTx) has profoundly changed the course of CKD and is associated with significant reductions in mortality and clinically relevant improvements in overall health and quality of life reported in kidney transplant recipients (KTR) compared to those treated with dialysis.^2-4^

Periodontitis is a multifactorial chronic inflammatory disease. It is associated with a dysbiotic biofilm and mediated by the host's immune response, potentially leading to permanent damage to periodontal tissues.^5,6^ This disease has been associated with several systemic conditions, including CKD.^7,8^ Considering that persistent inflammation is a leading cause of late graft loss in KTR,^9^ there is evidence that periodontal inflammatory burden may be associated with worsening graft function and increased risk of death due to the occurrence of adverse cardiovascular events in CKD.^10^

Periodontal therapy seeks to physically remove pathogenic biofilm and calculus, leading to a reduction in the immunoinflammatory response in periodontal tissues.^11^ Intervention modalities include surgical and nonsurgical periodontal therapy (NSPT). As the primary treatment of choice and widely considered the "gold standard" for the treatment of periodontitis, it includes oral hygiene guidelines, full-mouth scaling and root planning to remove supra- and subgingival biofilm and calculus.^12,13^ Previous studies have shown that NSPT can reduce systemic inflammation in CKD patients on dialysis.^14-16^

Despite the high prevalence of periodontitis in patients with CKD^17-19^ and the existence of evidence relating the altered presence of inflammatory mediators in patients with both diseases,^20-23^ to the best of our knowledge, no studies have investigated the effect of the clinical response to NSPT on changes in serum biomarkers in KTR. Thus, the hypothesis of this study is that NSPT, by reducing the local periodontal inflammatory burden, may have an additional effect on the serum biomarkers. Therefore, the aim of this study was to investigate the effect of NSPT on leukocyte count, fasting blood glucose, hemoglobin, hematocrit, creatinine, and uric acid in KTR.

## Methodology

### Study design

A prospective study was conducted with KTR at the University Hospital of the Federal University of Maranhão (HUUFMA), São Luís, State of Maranhão, Brazil. Initially, this study was approved by the Research Ethics Committee of the Federal University of Maranhão (CAAE: 55991616.6.0000.5087). All patients voluntarily agreed to participate by signing an informed consent form, after fully understanding of the collection objectives and methods employed during the study.

The study sample included patients of both sexes, aged ≥ 18 years, who underwent the KTx procedure at HUUFMA in 2016 and 2017, were immunosuppressed with tacrolimus regimen, and had at least six months of post-transplant follow-up. Non-inclusion criteria were patients who had renal graft loss, did not undergo post-transplant follow-up at HUUFMA, and/or died before data collection. Patients were excluded if they were hospitalized for infection after KTx, had a body mass index (BMI) ≥ 30 kg/m^2^, were edentulous, had orthodontic appliances, were pregnant, or had undergone periodontal therapy in the six months prior to the periodontal evaluation.

**Figure 1:**
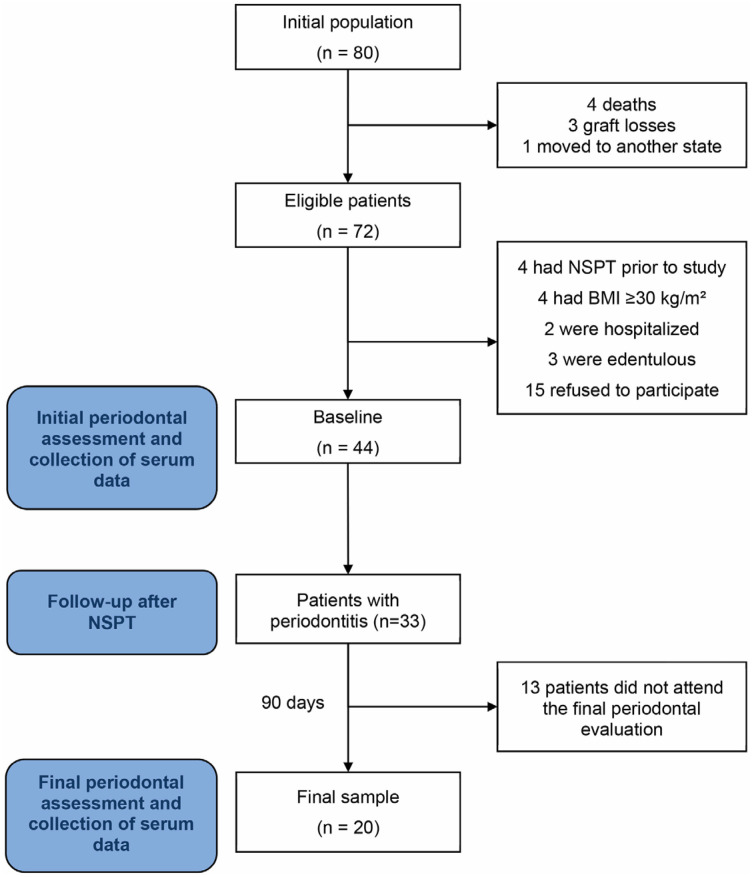
Flowchart of the screening process of study participants. NSPT: non-surgical periodontal therapy. BMI: Body mass index.

According to data from the Brazilian Association of Organ Transplantation, 33 KTx procedures were performed in Maranhão in 2016 and 47 in 2017.^24^ Of these 80 individuals, four died, three lost their kidney transplant, and one was transferred out of the state. Of the total 72 individuals eligible for this study, 44 met the eligibility criteria and agreed to participate in the study, undergoing an initial periodontal evaluation and serum data collection. Of these, the 33 patients with periodontitis underwent the NSPT intervention and were invited to return for a second evaluation in 90 days. However, 13 patients did not return for follow-up, resulting in 20 patients in the final study sample who underwent final periodontal evaluation and serum data collection (Figure 1). The *a priori* sample size calculation in G*Power version 3.1.9.6 (University of Kiel, Kiel, Germany) was based on the primary objective of estimating a moderate correlation coefficient (r = 0.65) between periodontal response and changes in serum biomarker levels after the follow-up period, considering a two-tailed test with alpha error equal to 0.05 and a power equal to 0.95. The minimum required sample size was 20 patients.

### Data collection

A semi-structured questionnaire was used to collect demographic variables and information on smoking habits (never smoked, former smoker). The underlying diseases of CKD (hypertension, diabetes, nephritis, and others) and comorbidities (hypertension, diabetes, and cardiovascular disease) were diagnosed by the hospital medical staff. Time of CKD diagnosis, time on dialysis, and time on KTx were collected by accessing medical records.

All serum biomarkers were measured in serum samples collected by a nurse technician on the same day with patients fasting for at least eight hours. All serum tests were processed in a standardized manner in the same laboratory at HUUFMA. Serum data were collected in two stages: time 0 (T0) represented the serum examination performed before the first periodontal evaluation (with a maximum interval of 7 days before the first periodontal evaluation), and time 1 (T1) was obtained from the serum examination performed after 90 days of NSPT. The following serum biomarkers were collected: fasting blood glucose (mg/dL), analyzed by the enzymatic method; hemoglobin (g/dL), hematocrit (%), and leukocyte count (thousand cells/mm^3^), analyzed by optical dispersion and cytochemistry with complementary microscopy; creatinine (mg/dL), analyzed by the Jaffe method; and uric acid (mg/dL), analyzed by the enzymatic Trinder method.

### Periodontal evaluation

The periodontal assessment was performed by one examiner (DLNS) who was trained prior to the start of clinical data collection to measure the intraclass correlation coefficient (ICC) for the periodontal probing depth (PPD, ICC = 0.86) and clinical attachment level (CAL, ICC = 0.88) measurements. Periodontal assessment was performed with a dental mirror no. 5 (Hu-Friedy®, Chicago, USA) and a Williams-type periodontal probe (Hu-Friedy®, Mgf. Co., Inc, Chicago, USA) under artificial light.

The following clinical parameters were assessed: PPD, the distance in millimeters between the gingival margin and the bottom of the periodontal pocket; and CAL, the distance in millimeters at the cementum-enamel junction and at the bottom of the periodontal pocket, with the highest probing values obtained at six sites on all teeth present except third molars.^25^ The bleeding on probing (BOP)^26^ score was also recorded based on the percentage of BOP-positive sites on all teeth. Patients were classified based on the presence of periodontitis and categorized into stages (1 to 4) according to the 2017 World Workshop on the Classification of Periodontal and Peri-Implant Diseases and Conditions criteria.^5^

### Nonsurgical periodontal therapy (NSPT)

NSPT was performed in a single session^13,27,28^ by one dentist (DLNS) in all patients and consisted of scaling and root planing, under local anesthesia if necessary, using Gracey-type periodontal curettes (Hu-Friedy®) and ultrasonic devices (piezoelectric Schuster®), followed by polishing with rubber cups and prophylactic paste and topical application of 1.23% acidulated phosphate fluoride for one minute. At the 90-day follow-up, the periodontal status was reassessed, followed by polishing and a new topical fluoride application. Based on the findings of the oral examination, patients could also receive scaling and root planing and/or other dental procedures, such as restorative treatment.

In accordance with standard protocol adopted by the hospital,^29^ during the two dental appointments, the patients were required to undergo antibiotic prophylaxis with 2 g of amoxicillin one hour before the appointment. The patients’ blood pressure was measured, with ideal values considered to be up to 140/90 mmHg. The patients were also instructed to rinse their mouths with 5 ml of 0.12% chlorhexidine gluconate (Periogard^®^) for one minute before the periodontal evaluation. At the end of both visits, patients received oral hygiene instructions to use a toothbrush with fluoridated toothpaste, using the modified Bass technique, and to use dental floss.

### Statistical analysis

Data analysis was performed using SPSS version 28.0 (IBM, Chicago, IL, USA). Descriptive analysis included frequencies, percentages, means, standard deviations (±SD), and standard errors of the mean (SEM). The Shapiro-Wilk test for normality was used to assess the distribution of continuous variables. Changes in serum and periodontal marker levels were evaluated using paired samples t-tests. Effect sizes for paired comparisons were reported as Cohen's d. In addition, delta was calculated to estimate the changes in periodontal clinical response and serum biomarkers, representing the differences between measurements taken 90 days after NSPT and before NSPT. Linear correlation analysis was used to estimate the strength and direction of the correlation between NSPT response and changes in serum variables.

Additionally, multivariate linear regression models were employed to examine the direct effect of changes in the percentage of CAL ≥3 mm on differences in serum biomarkers 90 days after NSPT, with adjustments made for potential confounding variables (age, sex, time since kidney transplantation, and diabetes). A 5% significance level was used for all tests.

## Results

A total of 20 patients (9 women and 11 men), ranging in age from 25 to 68 years, participated in this prospective study, with 35% identifying as former smokers. Former smokers had ceased smoking for a minimum of seven years. The most common underlying disease for CKD was nephritis (30%). Regarding comorbidities, it was observed that 75% of patients had hypertension, 30% had diabetes, and 15% had cardiovascular disease. The mean time of diagnosis of CKD was 97.5 ±56.8 months, while the time on dialysis and the time of KTx were 64.2 ±47.1 and 10.6 ±5.8 months, respectively. Half of the patients had stage 2 periodontitis (Table 1). In addition, the patients had at least nine teeth (mean 24.1 ±6.5 teeth). No patients lost teeth during the follow-up period.

**Table 1:** Distribution of demographic and clinical data of the study sample.

Variables	mean	±sd	range	n	(%)
**Sex**					
Female				9	(45.0)
Male				11	(55.0)
Age (years)	44.4	±14.3	(25–68)		
**Smoking history**					
Never smoked				13	(65.0)
Former smoker				7	(35.0)
**Underlying CKD disease**					
Diabetes				3	(15.0)
Hypertension				5	(25.0)
Nephritis				6	(30.0)
Other/ Unknown				6	(30.0)
**Comorbidities (%)**					
Diabetes				6	(30.0)
Hypertension				15	(75.0)
Cardiovascular disease				3	(15.0)
**CKD (months)**					
CKD diagnosis time	97.5	±56.8	(38–244)		
Time on dialysis	64.2	±47.1	(8–204)		
Time since KTx	10.6	±5.8	(6–28)		
**Number of teeth**	24.1	±6.5	(9–30)		
**Periodontitis**					
Stage 1				4	(20.0)
Stage 2				10	(50.0)
Stage 3–4				6	(30.0)

± sd: standard deviation, CKD: chronic kidney disease, KTx: kidney transplantation.

Table 2 shows the analysis of changes in periodontal and serum markers after NSPT. The data showed a significant reduction in the mean CAL (difference = −0.32, standard error = 0.08, P = 0.001, Cohen's d = −0.835), percentage of sites with PPD ≥3 mm (difference = −1.26, standard error = 0.43, P = 0.009, Cohen's d = −0.642), and PPD ≥4 mm (difference = −0.35, standard error = 0.10, P = 0.004, Cohen's d = −0.731). BOP values also significantly reduced after NSPT (difference = −2.92, standard error = 0.96, P = 0.007, Cohen's d = −0.675). Effect size measurements showed that NSPT had a greater effect on the mean CAL and percentage of sites with PPD ≥4 mm. Comparative analysis of serum biomarkers before and 90 days after NSPT showed no statistically significant differences in the total sample evaluated.

**Table 2:** Comparative analysis of periodontal variables and serum data at baseline and 90 days following nonsurgical periodontal therapy.

Variables	Before NSPT		90 days after NSPT		Difference (90 days after NSPT - before)		t	P	Effect size (Cohen's)
	mean	SEM	mean	SEM	mean	SEM			
Periodontal data									
Mean PPD (mm)	3.07	0.06	3.03	0.05	-0.04	0.02	-1.53	0.142	-0.342
Mean CAL (mm)	3.87	0.10	3.54	0.09	-0.32	0.08	-3.73	0.001*	-0.835
% of sites with PPD ≥3 mm	6.53	1.29	5.27	1.22	-1.26	0.43	-2.872	0.009*	-0.642
% of sites with PPD ≥4 mm	1.02	0.40	0.67	0.35	-0.35	0.10	-3.270	0.004*	-0.731
% of sites with CAL ≥3 mm	17.59	4.13	17.18	4.06	-0.40	1.06	-0.381	0.708	-0.085
% of sites with CAL ≥4 mm	6.16	2.24	5.66	1.92	-0.49	0.58	-0.844	0.409	-0.188
BOP	6.25	1.05	3.33	0.48	-2.92	0.96	-3.019	0.007*	-0.675
Serum data									
Fasting blood glucose (mg/dL)	99.54	5.44	107.08	9.12	7.53	9.45	0.797	0.436	0.178
Hemoglobin (g/dL)	12.81	0.52	13.08	0.47	0.27	0.22	0.829	0.417	0.185
Hematocrit (%)	39.67	1.48	40.73	1.34	1.05	1.10	0.958	0.350	0.214
Leukocytes (1,000 cells/mm³)	7.79	2.18	6.11	0.47	-1.68	2.08	-0.808	0.429	-0.180
Creatinine (mg/dL)	1.75	0.18	1.71	0.20	-0.03	0.06	-0.576	0.571	-0.128
Uric acid (mg/dL)	6.31	0.23	6.09	0.36	-0.22	0.23	-0.971	0.344	-0.217

SEM: standard error of the mean; NSPT: nonsurgical periodontal therapy; PPD: periodontal probing depth; CAL: clinical attachment level

BOP: bleeding on probing

* p <0.05, the paired Student's t-test

Analysis between the clinical periodontal response to NSPT and the change in serum biomarkers showed a direct correlation between the change in leukocyte count and mean CAL (r = 0.478, P = 0.033), percentage of sites with CAL ≥3 mm (r = 0.645, P = 0.002), and BOP (r = 0.663, P = 0.001). There was also a direct correlation between the change in uric acid and the percentage of sites with CAL ≥3 mm (r = 0.562, P = 0.010) (Table 3).

**Table 3:** Correlation analysis between clinical periodontal response and changes in serum biomarkers after NSPT.

Delta of serum variables	Clinical periodontal response to NSPT (90 days after NSPT – baseline)						
(90 days after NSPT – baseline)	Mean PPD	Mean CAL	% PPD ≥ 3 mm	% PPD ≥ 4 mm	% CAL ≥ 3 mm	% CAL ≥ 4 mm	BOP
	r	r	r	r	r	r	r
	(p value)	(p value)	(p value)	(p value)	(p value)	(p value)	(p value)
Fasting blood glucose	r=-0.274	r=0.011	r=0.011	r=-0.328	r=0.030	r=-0.093	r=-0.085
	p=0.242	p=0.965	p=0.962	p=0.158	p=0.899	p=0.696	p=0.722
Hemoglobin	r=0.153	r=-0.166	r=0.275	r=-0.255	r=0.279	r=0.008	r=0.160
	p=0.520	p=0.485	p=0.240	p=0.278	p=0.233	p=0.974	p=0.501
Hematocrit	r=0.214	r=-0.245	r=0.324	r=-0.326	r=0.408	r=0.070	r=0.159
	p=0.365	p=0.298	p=0.163	p=0.161	p=0.074	p=0.769	p=0.503
Leukocytes	r=-0.108	**r=0.478**	r=-0.019	r=-0.109	**r=0.645**	r=-0.341	**r=0.663**
	p=0.649	p=0.033*	p=0.936	p=0.648	p=0.002*	p=0.141	p=0.001*
Creatinine	r=-0.367	r=-0.144	r=-0.110	r=0.085	r=-0.120	r=-0.184	r=-0.205
	p=0.112	p=0.546	p=0.644	p=0.723	p=0.614	p=0.437	p=0.386
Uric acid	r=0.347	r=-0.297	r=0.265	r=0.217	**r=0.562**	r=0.281	r=-0.402
	p=0.083	p=0.203	p=0.258	p=0.359	p=0.010*	p=0.231	p=0.079

NSPT: nonsurgical periodontal therapy

PPD: periodontal probing depth

CAL: clinical attachment level

BOP: bleeding on probing

r: Pearson's correlation coefficient

* P <0.05.

Table 4 shows the direct effect of the changes in the percentage of CAL ≥3 mm on differences in serum biomarkers 90 days after NSPT, adjusted for age, sex (male), time since kidney transplantation, and diabetes. The coefficients of the model showed that the values of the differences in the percentage of CAL (after-before NSPT) had a direct positive effect on the differences in the levels of leukocytes (SRC = 0.678, 95% CI = 0.284 to 1.072, P 0.002) and uric acid (SRC = 0.643, 95% CI = 0.256 to 1.029, P 0.003), even after adjustment for potential confounding variables. Diabetes also had an effect on the difference in uric acid levels (SRC = 1.134, 95% CI = 0.041 to 2.226, P = 0.043). Creatinine level difference was affected by age, time since KTx, and diabetes. Furthermore, the multivariate models indicated that the differences in fasting blood glucose, hemoglobin, and hematocrit levels were not affected by the included variables.

**Table 4:** Multivariate linear regression models of the direct effect of the changes in the percentage of CAL ≥3 mm on differences in serum biomarkers 90 days after NSPT, adjusted for confounders variables.

Models	Direct effect on the difference in serum data		
	SRC	95% CI	p
Outcome: Fasting blood glucose			
Age	-0.266	-0.938 to 0.405	0.409
Male	-0.176	-1.519 to 1.165	0.782
Time since KTx	-0.091	-0.755 to 0.572	0.772
Diabetes	1.345	-0.145 to 2.835	0.073
Difference in % CAL ≥3 mm	-0.004	-0.531 to 0.523	0.986
Outcome: Hemoglobin			
Age	0.380	-0.309 to 1.070	0.257
Male	-0.395	-1.775 to 0.984	0.549
Time since KTx	0.068	-0.614 to 0.751	0.832
Diabetes	-0.168	-1.701 to 1.363	0.817
Difference in % CAL ≥3 mm	0.233	-0.308 to 0.775	0.370
Outcome: Hematocrit			
Age	0.346	-0.305 to 0.997	0.273
Male	-0.361	-1.664 to 0.940	0.561
Time since KTx	0.039	-0.604 to 0.683	0.897
Diabetes	0.003	-1.442 to 1.450	0.996
Difference in % CAL ≥3 mm	0.357	-0.154 to 0.868	0.156
Outcome: Leukocytes			
Age	0.094	-0.408 to 0.596	0.693
Male	-0.366	-1.370 to 0.637	0.446
Time since KTx	-0.065	-0.561 to 0.431	0.782
Diabetes	-0.660	-1.775 to 0.454	0.224
Difference in % CAL ≥3 mm	0.678	0.284 to 1.072	0.002*
Outcome: Creatinine			
Age	-0.637	-1.090 to −0.184	0.009*
Male	-0.174	-1.080 to 0.732	0.686
Time since KTx	0.759	0.310 to 1.207	0.003*
Diabetes	1.052	0.045 to 2.059	0.042*
Difference in % CAL ≥3 mm	0.124	-0.231 to 0.481	0.466
Outcome: Uric acid			
Age	-0.145	-0.636 to 0.347	0.538
Male	-0.612	-1.594 to 0.371	0.203
Time since KTx	0.465	-0.021 to 0.951	0.059
Diabetes	1.134	0.041 to 2.226	0.043*
Difference in % CAL ≥3 mm	0.643	0.256 to 1.029	0.003*

11 SRC: Standardized regression coefficient. 95% CI: 95% confidence interval. KTx: kidney transplantation. CAL: clinical attachment level.

* Significant direct effect on the level of the serum biomarker (p <0.05).

## Discussion

This study tested the hypothesis that NSPT, by reducing the periodontal inflammatory burden, could have a beneficial effect on serum levels of total leukocytes, fasting blood glucose, hemoglobin, hematocrit, creatinine, and uric acid in KTR, were that the reduction in the percentage of sites with CAL ≥3 mm and sites with BOP after NSPT is correlated with a decrease in leukocyte count, and the reduction in the percentage of sites with CAL ≥3 mm is correlated with a reduction in serum uric acid level. This suggests that NSPT may have an impact on reducing serum levels of leukocyte count and uric acid, with a beneficial effect on systemic health in KTR. A multivariate analysis adjusted for potential confounding variables supported these findings.

Leukocyte count is a strong biomarker of infection and systemic inflammation and correlates well with the host response to a variety of stimuli.^30,31^ Evidence suggests that an increase in serum leukocyte count is associated with a greater risk of developing the disease, and appears to be predictive of the risk of kidney failure in patients with early-stage CKD.^31,32^ In people with end-stage CKD, leukocyte function is altered, resulting in an impaired host response to infection.^33^ In our study, the improvement in periodontal attachment loss and reduction in periodontal inflammation after NSPT was directly correlated with the reduction in leukocyte count, suggesting a possible reduction in the level of systemic inflammation. These findings support the hypothesis that the patient's response to NSPT may have a positive impact on the patient's systemic health after KTx.

Considering that elevated leukocyte count may be a risk factor for several systemic diseases,^34^ including CKD, our results suggest that NSPT may benefit KTx patients by reducing the total number of circulating leukocytes in the blood. Studies have shown that the detection of signs of bloodstream infection, such as an elevated leukocyte count, is associated with a higher risk of death in patients following solid organ transplantation.^35-37^ These findings highlight the importance of periodontal infection control as an adjunct in monitoring the systemic status of KTR.

In periodontitis, increased leukocyte counts have been suggested to be mainly due to greater number of neutrophils, which act as the first line of defense and are part of the innate immune system. It is possible that these cells are recruited at higher levels during episodes of bacteremia in periodontitis.^30^ In the study by Azeez, Abdulhaq and Salih^27^ (2018), patients with periodontitis had a significantly increased leukocyte count compared to healthy individuals. In support of these findings, several studies have demonstrated the occurrence of statistically significant decreases in leukocyte counts after NSPT in the general population, with a consequent reduction in the risk of cardiovascular disease, particularly atherosclerosis.^28,38,39^ Therefore, our findings support the evidence that reducing the periodontal inflammatory burden may lead to a decrease in serum leukocyte levels.

Uric acid is a heterocyclic organic compound produced during the metabolism of purines in humans. It can exhibit potent antioxidant and free radical scavenging activities at physiological levels, but can also exhibit pro-inflammatory properties at higher levels.^40^ Elevation of serum uric acid levels is associated with diseases in which inflammation plays an important role in pathogenesis, such as metabolic syndrome, cardiovascular disease, and CKD.^41-43^ In CKD, a higher concentration of serum uric acid can lead to tubular injury, endothelial dysfunction, oxidative stress, and intrarenal inflammation,^44^ and is an important marker in managing the progression of this disease.^33^ In this study, we observed that the improvement in periodontal attachment loss after NSPT showed a direct correlation with the decrease in serum uric acid levels, suggesting a possible contribution to reducing the level of systemic inflammation in individuals after KTx.

Based on the role of uric acid in the inflammatory process, there may be an interaction between serum uric acid levels and the development and progression of periodontitis.^40^ There is evidence of a significant increase in uric acid levels in patients with periodontitis in the general population,^45-47^ and in KTR with periodontitis.^48^ In a previous study, we found that higher serum uric acid levels were associated with the occurrence of oral disease burden, a variable constructed from the presence of periodontitis and dental caries, in patients after KTx.^23^ This fact was corroborated by our current findings with the reduction of serum uric acid levels after 90 days of NSPT, reinforcing the hypothesis that NSPT may provide benefits to the systemic health of KTx patients.

In a previous study, we observed an association between periodontitis and hyperglycemia in KTX patients after crude and adjusted analyses,^22^ a finding supported by Shin and Mun^49^ (2023). This was motivation for the present study, so as to investigate the effect of NSPT on fasting blood glucose levels. However, no significant changes in this biomarker were found after 90 days of NSPT. For confirmation, we performed multivariate analyses adjusted for age, sex, diabetes, and time since KTx, in which the present study also observed no statistically significant change in fasting blood glucose levels after 90 days of NSPT. It is important to note that in the present study sample, 30% of the patients had diabetes, which can reduce the healing capacity and have a significant impact on the expression of periodontitis as well as the response to periodontal therapy.^14^ These factors may have contributed to the decrease in the effect of periodontal treatment on this biomarker in the present study. Considering that the bidirectional relationship between diabetes and periodontitis is well-established in the literature,^50-52^ this finding should be better explored in future studies with longer prospective follow-up. In addition, further studies should be conducted analyzing other biomarkers, such as glycated hemoglobin, insulin, and HOMA index, which better assess the patient's glycemic status.

A secondary finding of this study was that no correlation was identified between changes in creatinine and periodontal response following non-surgical periodontal therapy. Nevertheless, multivariate analysis demonstrated that creatinine was affected by age, time since kidney transplantation (KTx), and diabetes in the sample. Serum creatinine is a well-established marker for evaluating graft function recovery in kidney transplant patients.^53^ It is important to note, however, that serum creatinine exhibits considerable inter-individual variability, with potential differences observed according to factors such as sex, body measurements, medication use,^53,54^ and other variables, including diabetes.^55^ The aforementioned evidence provides an explanation for the present findings.

In general, there was a reduction in the mean values of all periodontal parameters after scaling and root planing, with statistically significant changes in the percentage of sites with PPD ≥3 mm and ≥4 mm and in BOP. These are the periodontal parameters most closely related to periodontal disease activity and which better reflect the impact of inflammation on periodontal tissues,^5^ demonstrating the efficacy of the NSPT performed and its impact on local inflammation. In addition, the results also suggest a potential beneficial effect on the systemic health of these patients.

To the best of our knowledge, this is the first study to investigate the influence of NSPT on serum levels of leukocyte count, fasting blood glucose, hemoglobin, hematocrit, creatinine, and uric acid in KTR. A key strength of this research was the observed the positive effect of NSPT on serum levels of leukocyte count and uric acid, as indicated from correlation analyses between changes in periodontal variables and serum biomarkers after treatment. In addition, according to the Kidney Disease Improving Global Outcomes Transplant Work Group,^56^ the first months after KTx are the period with the highest risk of acute graft rejection and infection, during which the patient uses higher doses of immunosuppressive drugs, with the peak period of immune-mediated complications occurring at 3 months. Thus, the best time for dental treatment is during the period of stability, which is approximately 6 months after transplantation.^57^ As such, this cutoff point was used as the inclusion criterion. Therefore, data collection and periodontal intervention were performed during a period of greater stability of the patient's health status.

Another strength of the study was the reduction in both the risk of bias and in the potential confounding effect of different drug regimens on the patients. To achieve this, patients with a BMI ≥ 30 kg/m^2^ were excluded, and all patients were subjected to immunosuppressive treatment with tacrolimus. In addition, models were used to support the main findings after adjusting for confounding variables (age, sex, time since KTx, diabetes). However, a significant limitation of this study was its small sample size, which may have reduced the power to detect other associations. Despite this, all patients who underwent KTx in Maranhão in 2016 and 2017, met the eligibility criteria, consented to participate, and attended both appointments were included in the analysis. Therefore, it is recommended to develop future studies with larger samples and longer follow-up, in addition to investigating other systemic health biomarkers, to further deepen the investigation of positive systemic effects associated with periodontal therapy.

## Conclusion

A good clinical periodontal response to NSPT appears to be associated with decreased serum levels of leukocytes and uric acid. These findings suggest that the management of periodontal inflammation may have a beneficial effect on systemic health in KTR.

## Data availability statement

The datasets generated during and/or analyzed during the current study are available from the corresponding author on reasonable request.
